# Micronutrients Potential of Underutilized Vegetables and Their Role in Fighting Hidden Hunger

**DOI:** 10.1155/2020/9408315

**Published:** 2020-03-21

**Authors:** James S. Chacha, Henry S. Laswai

**Affiliations:** Department of Food Technology, Nutrition and Consumer Sciences, College of Agriculture, Sokoine University of Agriculture, P.O. Box 3006, Morogoro, Tanzania

## Abstract

**Background:**

Innumerable underutilized vegetable (UV) species have been utilized as food and as folklore medicine since time immemorial. Such vegetables have been part and parcel of the food dishes, especially to the ancient rural and periurban dwellers. However, researchers and agricultural scientists have given little or no attention to such vegetables, as to what constitutes their potentials in curbing hidden hunger. To achieve the global Sustainable Development Goals, Tanzania needs to address the issue of food insecurity through the use of not only grains, fruits, and edible insects but also through embracing the UVs. The overall objective of this study was to screen the indigenous vegetables with nutritional and health claims from communities in Kilimanjaro and Morogoro regions.

**Methods:**

Quantitative data were obtained by conducting laboratory nutrient and antinutrients composition analyses as per standard Association of Official Analytical Chemists (AOAC) methods. This was carried out to determine the moisture content, micronutrient, and antinutrients composition of the selected UVs.

**Results:**

The nutritional and medicinal claims of the selected UVs mentioned during interviews were validated by data obtained from laboratory nutrient and antinutrients composition analyses. Chemical analyses revealed that vitamin A, B_1_, B_2_, B_3_, and C contents ranged from 2.50-6.67, 18.94-182.95, 0.18-0.76, 0.09-0.43, and 46.52-198.08 mg/100 g, respectively. Minerals, on the other hand, Ca, Fe, Mg, and Zn contents ranged from 60.28-421.03, 4.28-21.05, 191.12-1151.91, and 4.28-21.10 mg/100, respectively. Moisture content, oxalates, and phytates contents ranged from 78.59-95.49%, 1.28-3.15, and 1.64-6.18 mg/100 g, respectively.

**Conclusion:**

The findings from the study added credence to the selected UVs that they are rich sources of micronutrients and crucial in daily human diet to curb hidden hunger.

## 1. Background

Food insecurity and malnutrition affect much of the world's population [1]. Approximately two billion people, representing every country on earth, are estimated to suffer from micronutrient deficiencies that make them more susceptible to disease, creating a significant obstacle to economic growth [2]. About 239 million of the people in sub-Saharan Africa are affected by chronic undernutrition [3]. According to Ezzati et al. [4], the low consumption of vegetables and fruits is among the top ten risk factors for mortality. The diets of people in both urban and rural areas are mainly cereal-based resulting in poor diets with increased prevalence of nutritional deficiency disorders [5].

Micronutrient dietary deficiencies that lead to nutritional disorders are still common in Tanzania [6]. According to Weinberger and Msuya [7], it is important that the rich sources of such nutrients are identified and promoted for daily consumption. Wild vegetables in particular play significant roles in the livelihood of many communities in the developing countries as food and medicinal [8]. They contain micronutrients that aid in promoting immunity against infections and providing food security for the people [9].

Unfortunately, little has been done regarding the UVs' nutritional and medicinal use. Regarding the UVs' nutritional and medicinal properties, many of them are still scientifically unexplored and grow wildly [10]. This explains why they are generally uncultivated and underutilized [11]. According to Rita et al. [12], if the UVs could be properly harnessed and utilized, they could be an antidote to food insecurity and malnutrition in Tanzania. Therefore, this study aims at exploring the micronutrient composition of the selected UVs and their role in fighting hidden hunger.

## 2. Methods

### 2.1. Study Areas

The study was conducted in 2017 in Morogoro and Kilimanjaro regions, Tanzania. These regions were selected as an eastern and northern zonal representation of Tanzania regions participating in agriculture, respectively. Two districts were selected from each region: Kilosa district which lies between latitudes 5°55′ and 7°53′S and longitudes 36°30′ and 37°30′E and within an altitude of 200 to 700 m above the sea level, covering a total area of 14,918 km^2^; Mvomero district found between latitudes 05°80′ and 07°40′S and between longitudes 37°20′ and 38°05′E, covering a total area of 7,325 km^2^; Rombo district found between latitudes 2°50′ and 3°23′S and longitude 37°15′ and 37°41′E and covers an area of 1,442 km^2^ 14; and Hai district located between 2°50′S and 3°29′S and longitude 30°30′E and 37°10′E, covering an area of 13,000 km^2^.

Kilosa district has a bimodal rainfall distribution, with early rains starting from October to December while the latter rains periods between January and May. The highest parts of the district get annual rainfall of 1,000-1,600 mm whereas the central and southern parts an average of 800-1,400 mm. Temperature varies between 15 and 32°C with mean annual temperature of 25°C. The main economic activity carried out at Kilosa district is agriculture (including crop farming and livestock keeping). Mvomero district has temperature range from 18 to 30°C, with annual rainfall from 600 to 1,000 mm. The area experiences bimodal rainfall pattern where long rains are from March to the end of May and short rains occur from October to December. The dry seasons are from June to August and January to March. The district economy depends mainly on agriculture.

Rombo district has volcanic soils, with the rainfall pattern being bimodal, short rains from November to December and long rains from March to May. Rainfall ranges from 1,000 mm to 2,000 mm on average and varies with elevation while temperature ranges from 18° to 28°C. The natives depend on subsistence and small scale farming, livestock keeping, and some depend on retail business. Hai district experiences two main rain seasons: the long rain season which begins in March and ends in June and the short rain season that starts in November and ends in December. The area has soils that are mainly alluvial and volcanic in nature and experiences a temperature of 20°C and an average annual rainfall of 700 mm. Most people earn their living through farming, livestock keeping, and trade.

### 2.2. Research Design

The study design was cross-sectional and was split into two components: qualitative and quantitative. This particular study covers the quantitative component (experimental laboratory work) which was carried out to identify the nutrients and antinutrients present in the vegetables. After the interviews, from the study sites, samples of the edible portions of the selected underutilized vegetables (the leaves) were collected and taken to the laboratory at Sokoine University of Agriculture for analysis.

### 2.3. Data Collection Methods

Leaves were harvested, and quantitative data was obtained through laboratory analyses. Moisture content determination was done using the oven drying method as per AOAC procedures [13]. Mineral contents were determined using atomic flame emission spectrophotometer (AA-6200 Shimadzu Corp, Kyoto Japan) as per the AOAC procedure [13]. Beta-carotene was determined using standard AOAC Method 2005.07 [14]. Vitamin B_1_ was determined by spectrophotometry as per AOAC Method 942.23 [14], vitamin B_2_ by fluorometry using AOAC Method 970.65 [14], vitamin B_3_ by colorimetric method as described by Deutsch [15], and vitamin C content using 2,6-dichorophenol indophenol method as per AOAC Method 967.21 [14]. Phytate content was determined by the method adopted by Davis [16], while oxalate content was determined using AOAC method 974.24.

### 2.4. Data Analysis

The test data generated from the laboratory analyses were subjected to one-way analysis of variance (ANOVA) to determine the significant differences in means, using Statistical Package for Social Sciences (SPSS) software version 16.0. The Duncan post hoc homogeneity tests were calculated to separate the significant attributes. Results were expressed as means ± standard deviation.

## 3. Results and Discussion

### 3.1. Sunga (Bitter Lettuce, *Launea cornuta*)

#### 3.1.1. Nutritional and Health Potentials of *L*. *cornuta*

**Figure 1:**
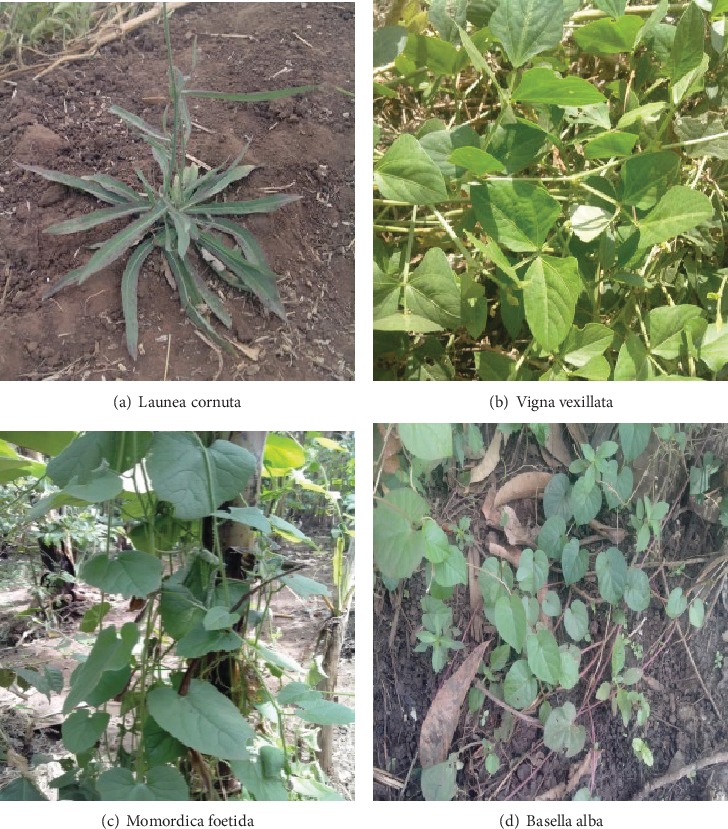
Selected UVs from Morogoro and Kilimanjaro regions.

Nutritionally, *L. cornuta* (Figure 1(a)) was claimed to be a rich source for vitamins, though the respondents were not able to point out the exact vitamin. They also claimed that the vegetable provides strength (energy) and increases appetite as supported by Muriira et al. [17], a fact that was attributed to its bitterness and that an individual usually feels hungry after consuming it. The interview reports were supported by laboratory analyses, which showed the presence of considerable amounts of calcium, iron, magnesium, zinc, vitamins A, B_1_, B_2_, B_3_, and C to be 60.29, 301.56, 6.05, 3.84, 25.22, 0.24, 0.1, and 120.88 mg/100 g, respectively (Table 1). The findings agree with a study by Lyimo et al. [18] who found the vegetable as a rich source of minerals and vitamins.

Medicinally, the vegetable was reported to provide cure for a number of diseases including malaria and typhoid, which agrees with a study by Musila et al. [19]. The curing attribute is mainly attributed to bitter juice/sap contained in the vegetable.

### 3.2. Kikundembala (Wild Cowpea, *Vigna vexillata*)

#### 3.2.1. Nutritional and Medicinal Potentials of *V*. *vexillata*

The claims by respondents that the plants provide vitamins were collaborated by the laboratory experiments conducted which revealed that *B. alba* is a rich source of vitamins, whereby the vitamin contents specifically vitamins A, B_1_, B_2_, B_3_, and C were found to be 2.57, 18.94, 0.18, 0.09, and 136.71 mg/100 g, respectively. On the other hand, the mineral contents specifically calcium, iron, magnesium, and zinc were found to be 85.28, 4.28, 191.12, and 4.28 mg/100 g, respectively (Table 1).

The findings are in line with a study done by Kumar and Kumar [20] whereby it was observed that the vegetable is a good source of micronutrients among them iron and zinc. It was also claimed to increase strength, increase blood levels (a claim that can be attributed to the iron components of the leaves), and cure eye problems and hernia (roots boiled and its water drank, thought to be contributed by its vitamin A content). Moreover, pains due to menstrual flow can be relieved using the vegetable, the healing virtue being attributed to the claim that no insecticides or pesticides are applied on the vegetable.

### 3.3. Mokiki (Bitter Cucumber, *Momordica foetida*)

#### 3.3.1. Nutritional and Medicinal Potentials of *M. foetida*

*M*. *foetida* (Figure 1(c)) was claimed to increase strength, appetite, and blood. It was reported that the leaves of the plant can be prepared and eaten alongside any other vegetable and or food. Laboratory analyses revealed the presence of mineral contents specifically calcium, iron, magnesium, and zinc was found to be 421.03, 21.05, 1,151, and 21.1 mg/100 g, respectively (Table 1). Also, vitamin contents specifically vitamins A, B_1_, B_2_, B_3_ and C were found to be 5.5, 58.34, 0.76, 0.12, and 46.52 mg/100 g, respectively. The presence of vitamins is thought to contribute to the appetite-increase-role of the UV.

Medicinally, *M*. *foetida* relieves cough/flu, whereby its leaves are chewed raw or the leaves are plucked, wrapped in banana leaves, and put on fire to be heated for a while; thereafter, it is removed and chewed. A mixture of *M. foetida*, “Ngolowo” and “Ibangasa” constitutes a liquid which is used as medicine against smallpox. The mode of treatment involves drinking of the mixture by the sick child who is then covered by a blanket for a sleep. A lot of sweating takes place, a procedure believed to indicate that the medicine is carrying out its functions! Also, it was reported that the UV helps in relieving diarrhoea and malaria as well as in removing toxins from the body. Moreover, *M*. *foetida* has scientifically been demonstrated to contain hypoglycaemic activity [21].

### 3.4. Inyiri (Malabar Spinach, *Basella alba*)

#### 3.4.1. Nutritional and Medicinal Potentials of *B. alba*

*B. alba* (Figure 1(d)) was nutritionally claimed to be a good source of vitamins and minerals. The claims were supported by the laboratory analysis conducted which revealed *B. alba* as a rich source of minerals and vitamins. The mean mineral contents, specifically calcium, iron, magnesium, and zinc, were found to be 134.11, 13.4, 524.5, and 13.4 mg/100 g, respectively. Also, the mean vitamin contents, specifically vitamins A, B_1_, B_2_, B_3_, and C were 6.67, 182.95, 0.54, 0.43, and 198.08 mg/100 g, respectively (Table 1).

Medicinally, the vegetables were claimed to boost blood supply and increase appetite. These effects were attributed to their iron and vitamins contents, respectively. They prevent constipation due to the fibrous nature, relieve ulcerative pains, and prevent constipation in animals, for instance dogs. The claims are in fair agreement to the laboratory chemical analyses, which revealed *B*. *alba* as a rich source of iron, an important component in fighting anaemia. Also, Kumar et al. [22] support that *B. alba* leaves are used for the treatment of anaemia in women and other diseases including hypertension, malaria, coughs, and colds.

## 4. Conclusion

The numerous varieties of UVs in Morogoro and Kilimanjaro regions offer potential sources of micronutrients; nevertheless, just like other plants, they contain varying levels of antinutrients, especially phytates and oxalates. Consumption of the UVs will definitely benefit the communities in relation to reducing hidden hunger, thus ensuring food and nutritional security in Tanzania.

### 4.1. Recommendations

There is an urgent need for promotion of consumption of UVs because of their nutritional significance. Moreover, given the benefits from this study, practical methods need to be developed to acquire the seeds of the selected UVs for domestication to ensure a wider adoption on a sustainable basis. Furthermore, there is a need to study effects of their preparation and cooking in order to ascertain the safety of their consumption, due to the presence of some antinutrients.

## Figures and Tables

**Table 1:** Micronutrient and antinutrient content of selected UVs.

Vegetable parameter	*Momordica foetida*	*Vigna vexillata*	*Launea cornuta*	*Basella alba*
Moisture content (%)	95 ± 0.00^c^	78 ± 1.41^a^	84 ± 1.41^b^	92.5 ± 0.71^c^
Minerals (mg/100 g)				
Ca	421.03 ± 3.61^c^	85.28 ± 4.67^ab^	60.29 ± 4.26^a^	134.11 ± 17.16^b^
Fe	21.05 ± 1.77^c^	4.28 ± 0.23^a^	6.04 ± 0.43^a^	13.4 ± 1.70^b^
Mg	1,151.91 ± 49.47^c^	191.12 ± 19.64^a^	301.56 ± 21.31^a^	524.5 ± 121.08^b^
Zn	21.1 ± 1.84^c^	4.28 ± 0.23^a^	6.05 ± 0.42^a^	13.4 ± 1.70^b^
Vitamins (mg/100 g)				
Beta carotene	5.5 ± 0.77^b^	2.5 ± 0.40^a^	3.84 ± 0.50^a^	6.67 ± 0.30^b^
B vitamins				
B_1_	58.34 ± 2.67^a^	18.94 ± 0.69^a^	25.22 ± 0.60^a^	182.95 ± 47.76^b^
B_2_	0.76 ± 0.06^c^	0.18 ± 0.01^a^	0.24 ± 0.02^a^	0.54 ± 0.12^b^
B_3_	0.12 ± 0.02^a^	0.09 ± 0^a^	0.1 ± 0.02^a^	0.43 ± 0.06^b^
C	46.52 ± 3.70^a^	136.71 ± 35.84^b^	120.88 ± 11.26^ab^	198.08 ± 47.46^b^
Antinutrients (mg/100 g)				
Oxalate	2.74 ± 0.75^a^	1.28 ± 0.71^a^	3.15 ± 0.81^a^	1.34 ± 0.13^a^
Phytates	6.18 ± 0.04^c^	1.64 ± 0.06^a^	1.74 ± 0.05^a^	4.31 ± 0.52^b^

Values are expressed as means ± SD (*n* = 2). Mean values with different superscripts in a column are significantly different (*P* < 0.05).

## Data Availability

The datasets used and/or analysed during the current study are available from the corresponding author on reasonable request.

## Abbreviations

AOAC: Association of official analytical chemists
UVs: Underutilized vegetables.
